# Challenges and strategies for robotic portal anatomical resection in patients with thoracic deformities

**DOI:** 10.1093/jscr/rjag153

**Published:** 2026-06-19

**Authors:** Ryusuke Sumiya, Shinsuke Uchida, Mariko Fukui, Takeshi Matsunaga, Yukio Watanabe, Aritoshi Hattori, Kazuya Takamochi, Kenji Suzuki

**Affiliations:** Department of General Thoracic Surgery, Juntendo University School of Medicine, 2-1-1, Hongo, Bunkyo, Tokyo, 113-8421, Japan; Department of General Thoracic Surgery, Juntendo University School of Medicine, 2-1-1, Hongo, Bunkyo, Tokyo, 113-8421, Japan; Department of General Thoracic Surgery, Juntendo University School of Medicine, 2-1-1, Hongo, Bunkyo, Tokyo, 113-8421, Japan; Department of General Thoracic Surgery, Juntendo University School of Medicine, 2-1-1, Hongo, Bunkyo, Tokyo, 113-8421, Japan; Department of General Thoracic Surgery, Juntendo University School of Medicine, 2-1-1, Hongo, Bunkyo, Tokyo, 113-8421, Japan; Department of General Thoracic Surgery, Juntendo University School of Medicine, 2-1-1, Hongo, Bunkyo, Tokyo, 113-8421, Japan; Department of General Thoracic Surgery, Juntendo University School of Medicine, 2-1-1, Hongo, Bunkyo, Tokyo, 113-8421, Japan; Department of General Thoracic Surgery, Juntendo University School of Medicine, 2-1-1, Hongo, Bunkyo, Tokyo, 113-8421, Japan

**Keywords:** robotic surgery, thoracic deformity, Haller index

## Abstract

Anatomical resection using a robotic approach in patients with thoracic deformities presents several challenges, including port placement. We present two patients with thoracic deformities—one without (Case 1) and one with (Case 2) specific port placements—focusing on difficulties such as limited visibility and restricted motion of the robotic arm.

## Introduction

Anatomical resection in patients with thoracic deformities such as pectus excavatum and straight-back syndrome using the robotic approach presents several challenges, including port placement. Herein, we present a knack and pitfalls of robotic portal right-side anatomical resection in patients with thoracic deformities through two cases.

## Materials and surgical technique

**Figure 1:**
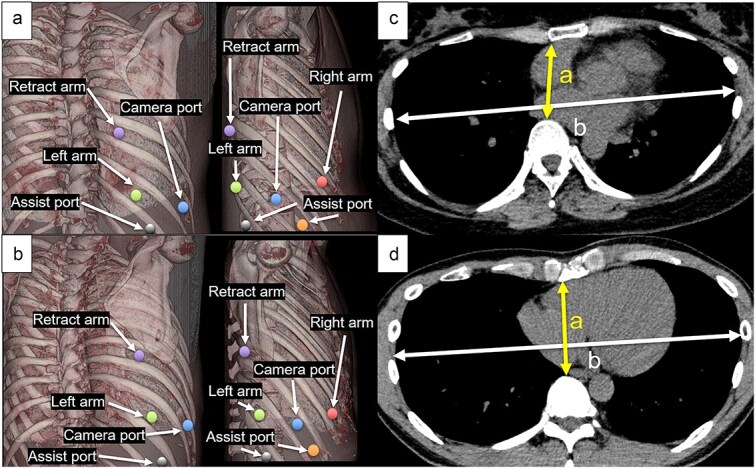
Representative schema of port placements of Case 1 (a) and Case 2 (b), and computed tomography images of Case 1 (c) and Case 2 (d). (a, b) purple: Retracting arm, green: Left arm, blue: Camera port; red: Right arm, orange and black: Assist port. (c, d) a: Minimum anterior–posterior diameter, b: Maximum transverse diameter.

**Figure 2:**
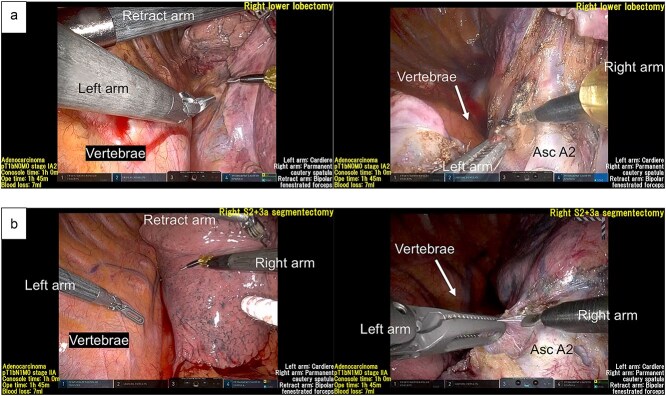
Representative intraoperative images of Case 1 (a) and Case 2 (b).

First, we describe the general port placement at our institute [1, 2]. Patients were placed in the left lateral decubitus position. At our institute, three 8-mm robotic ports were placed in the sixth, eighth, and ninth intercostal spaces, and one 12-mm robotic port was placed in the sixth intercostal spaces. The procedure was performed using the da Vinci Xi robot (Intuitive Surgical, Sunnyvale, Calif, USA). The robotic instruments included a spatula (right arm), Cadiere forceps (left arm), and a double fenestrated grasper (retracting arm). The retraction arm was generally placed at least 4 cm away from the vertebrae, and the distance between the ports was at least 6 cm. Case 1 involved a 41-year-old woman who underwent a right lower lobectomy for lung cancer using the general port placement (Fig. 1a). Case 2 involved a 38-year-old man who underwent a right S2 + 3a segmentectomy for lung cancer using a special port arrangement (Fig. 1b). Although neither patient had been diagnosed with pectus excavatum, the Haller index was 4.5 in Case 1 (Fig. 1c) and 3.8 in Case 2 (Fig. 1d). When the operation was performed with the general port placement, as in Case 1, the protruding vertebra obstructed the surgeon’s view, especially the pulmonary hilum (Video 1). In addition, the motion of the retraction arm was definitively limited by the vertebrae (Fig. 2a). Therefore, in Case 2, the camera port was shifted slightly to the ventral side, and the retraction arm was placed 8 cm from the vertebrae (Video 2). Although the distance between the ports was reduced to avoid interference between the retraction arm and the other arms, the operation was performed more comfortably than in Case 1 (Fig. 2b).

## Discussion

**Figure 3:**
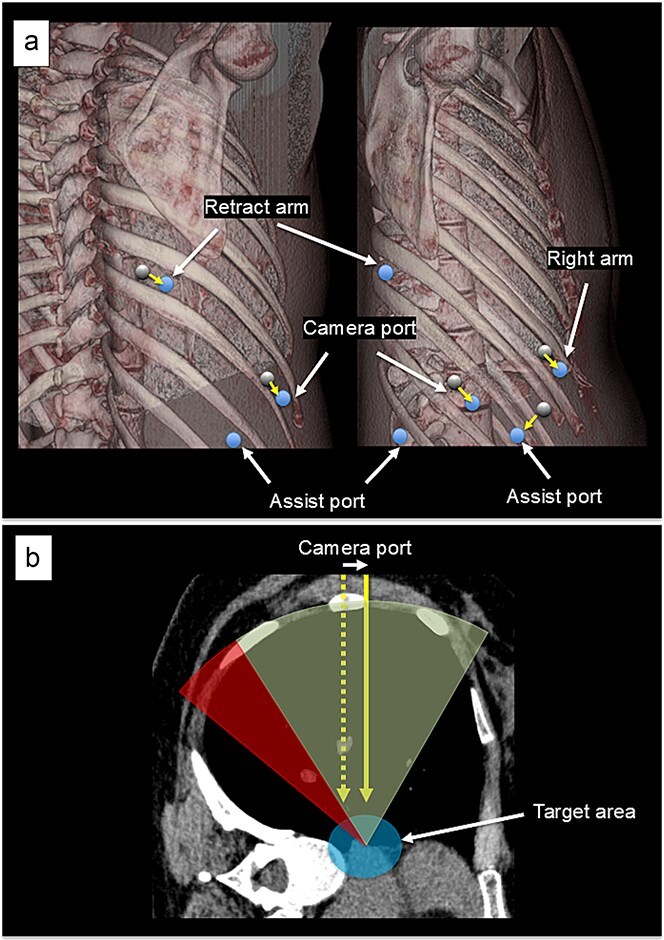
Schemas of the modification of port placement (a) and an overview (b). (a) The black circle indicates the conventional port recommendation and the blue circle indicates the modified port placement. (b) The blue circle indicates the target area. The green region indicates the acceptable area for port placement in cases of thoracic deformity, and the red region indicates area that are not acceptable for port placement in cases of thoracic deformity, but which are acceptable for patients without thoracic deformity. The yellow solid arrow indicates the modified camera port and the yellow dotted arrow indicates the camera port in a typical case.

When considering anatomical lung resection on the left side, the retraction arm should be positioned as far as possible from the vertebrae to avoid contact between the retraction arm and the aorta. Likewise, in the right-sided approach for the patients with thoracic deformities, appropriate port placement should be ensured—specifically, the vertical line made by the camera port and the mediastinal plane lie anterior to the vertebral body. This ensures sufficient space between the vertebral body and the retraction arm, even if it requires reducing the inter-port distance to <6 cm. This is particularly important in anatomical resection of the right upper and lower lobes, which they often require a dorsal approach. As in Case 1, when approaching patients with thoracic deformities without specific preparation, several difficulties may be encountered, such as poor visibility and significant motion limitations of the robotic arm. However, in Case 2, because the port placements were shifted to the ventral side, the robotic approach was performed comfortably without contacting the vertebra (Fig. 3).

The Haller index is a major diagnostic indicator of pectus excavatum [3]. Beyond its original use, the Haller index may have potential for screening patients with thoracic deformities [4]. However, because the Haller index only indicates the ratio of the short to the long diameters of the thoracic cavity, it does not account for actual dimensions. Indeed, although Case 2 had a lower Haller index, the actual short and long diameters were greater than those of Case 1. In this study, in addition to port placement, the patient’s thoracic dimensions in Case 2 may have allowed for a simpler operation. Furthermore, the type of anatomical resection performed is another limitation. A segmentectomy of the right lower lobe was performed in Case 1, whereas a segmentectomy of the right upper lobe was performed in Case 2. This difference may have affected the difficulty of the surgical procedure, and further studies are needed to confirm this finding. In addition, because this study examined only right-sided anatomical resections, it is necessary to assess left-sided cases in future investigations.

## Conclusion

When considering robotic right-sided anatomical resection in patients with thoracic deformities, appropriate port placement should be performed.

## Supplementary Material

Video_case_1_rjag153

Video_case_2_rjag153

## Supportive information

Robotic portal right lower lobectomy in Case 1 (Video 1) and right upper lobectomy in Case 2 (Video 2) are summarized.
